# USING CHRONIC DISEASE SELF-MANAGEMENT TO ENHANCE PATIENT-PROVIDER PARTNERSHIPS

**DOI:** 10.1093/geroni/igz038.2908

**Published:** 2019-11-08

**Authors:** Brea Case, Angela M Zell, Joan Ilardo

**Affiliations:** 1 Michigan State University College of Human Medicine, Grand Rapids, Michigan, United States; 2 Michigan State University College of Human Medicine, East Lansing, Michigan, United States

## Abstract

The Partners in Aging Strategies and Training (PAST) project employed a bilateral approach to educate both healthcare professionals and consumers. Our theory is that improved health outcomes are attained by teaching healthcare providers and consumers how to engage better with each other, especially when consumers use the skills learned in community-based programs, such as self-management and healthy lifestyle choices. PAST activities provided an integrated educational program for healthcare providers and older adult patients, their families and caregivers to learn skills that enhance their ability to form productive patient-provider partnerships. We used three types of training: 1) multi-disciplinary health professions and primary care provider continuing-education face-to-face workshops and webinars; 2) older adult patient and caregiver workshops, resource materials; and 3) reverse marketing comprised of sending information to physicians whose patients attended a workshop that included the topics covered in the workshops and the patients’ three- to six-month action goals. We found that physicians who attended the grand rounds presentations were very receptive to the ‘nuts and bolts’ approach to things like doing a quick mobility assessment, effectively communicating with patients, health literacy, and referring patients to community-based non-medical services and supports. We conducted seven types of evidence-based workshops. Over 90% of participants gave permission to send a letter to their physician to tell them they attended the workshop. We used pre-post confidence scales based on each workshop’s learning objectives to measure changes in workshop participants’ self-management confidence. There was positive change in confidence for all seven workshops.

